# Heterogeneous SSTR2 target expression and a novel *KIAA1549*::*BRAF* fusion clone in a progressive metastatic lesion following ^177^Lutetium-DOTATATE molecular radiotherapy in neuroblastoma: a case report

**DOI:** 10.3389/fonc.2024.1408729

**Published:** 2024-09-11

**Authors:** Se Whee Sammy Park, Susanne Fransson, Fredrik Sundquist, Joachim N. Nilsson, Per Grybäck, Sandra Wessman, Jacob Strömgren, Anna Djos, Henrik Fagman, Helene Sjögren, Kleopatra Georgantzi, Nikolas Herold, Per Kogner, Dan Granberg, Mark N. Gaze, Tommy Martinsson, Kasper Karlsson, Jakob J. E. Stenman

**Affiliations:** ^1^ Department of Oncology-Pathology, Karolinska Institutet, Stockholm, Sweden; ^2^ Department of Laboratory Medicine, Sahlgrenska Academy, University of Gothenburg, Gothenburg, Sweden; ^3^ Department of Urology, Karolinska University Hospital, Stockholm, Sweden; ^4^ Department of Women’s and Children’s Health, Karolinska Institutet, Stockholm, Sweden; ^5^ Department of Medical Radiation Physics and Nuclear Medicine, Karolinska University Hospital, Stockholm, Sweden; ^6^ Department of Molecular Medicine and Surgery, Karolinska Institutet, Stockholm, Sweden; ^7^ Department of Pathology and Cancer Diagnostics, Karolinska University Hospital, Stockholm, Sweden; ^8^ Department of Pediatric Radiology, Karolinska University Hospital, Stockholm, Sweden; ^9^ Clinical Genetics and Genomics, Sahlgrenska University Hospital, Gothenburg, Sweden; ^10^ Department of Pediatric Oncology, Karolinska University Hospital, Stockholm, Sweden; ^11^ Department of Breast, Endocrine Tumors and Sarcomas, Karolinska University Hospital, Karolinska Institutet, Stockholm, Sweden; ^12^ Department of Molecular Medicine and Surgery, Karolinska University Hospital, Karolinska Institutet, Stockholm, Sweden; ^13^ Department of Oncology, University College London Hospitals National Health Service (NHS) Foundation Trust, London, United Kingdom; ^14^ Department of Pediatric Surgery, Karolinska University Hospital, Stockholm, Sweden

**Keywords:** LuDO-N clinical trial, ^177^Lutetium-DOTATATE, molecular radiotherapy, neuroblastoma, tumor heterogeneity, *KIAA1649::BRAF* gene fusion, case report

## Abstract

In this case report, we present the treatment outcomes of the first patient enrolled in the LuDO-N trial. The patient is a 21-month-old girl diagnosed with high-risk neuroblastoma (NB) and widespread skeletal metastasis. The patient initially underwent first-line therapy according to SIOPEN HRNBL-1 but was switched to second-line treatments due to disease progression, and she was finally screened for enrollment in the LuDO-N trial due to refractory disease. Upon enrollment, the patient received two rounds of the radiolabeled somatostatin analogue lutetium-177 octreotate (^177^Lu-DOTATATE), which was well tolerated. A dosimetry analysis revealed a heterogeneous uptake across tumor lesions, resulting in a significant absorbed dose of 54 Gy in the primary tumor, but only 2 Gy at one of the metastatic sites in the distal femur. While the initial treatment response showed disease stabilization, the distal femoral metastasis continued to progress, leading to the eventual death of the patient. A tissue analysis of the biopsies collected throughout the course of the disease revealed heterogeneous drug target expression of somatostatin receptor 2 (SSTR2) across and within tumor lesions. Furthermore, genomic profiling revealed a novel *KIAA1549*::*BRAF* fusion oncogene amplification in the distal femoral metastasis at recurrence that might be related with resistance to radiation, possibly through the downregulation of SSTR2. This case report demonstrates a mixed response to molecular radiotherapy (MRT) with ^177^Lu-DOTATATE. The observed variation in SSTR2 expression between tumor lesions suggests that heterogeneous target expression may have been the reason for treatment failure in this patient’s case. Further investigation within the LuDO-N trial will give a more comprehensive understanding of the correlation between SSTR2 expression levels and treatment outcomes, which will be important to advance treatment strategies based on MRT for children with high-risk NB.

## 1 Introduction

Neuroblastoma (NB) is a malignant neuroendocrine tumor that arises from the developing sympathetic nervous system (1). The clinical course varies widely, with some patients having a favorable prognosis, whereas about half of the patients have high-risk neuroblastoma (HR-NB) (2). The current treatment protocols for HR-NB, such as SIOPEN HR-NBL2 (NCT04221035), include intensive induction chemotherapy, myeloablative consolidation chemotherapy with stem cell rescue, and local control via surgery and radiation as well as maintenance treatment with anti-GD2 antibodies and retinoic acid (3–6). Despite the multimodal treatment, the mortality rate for HR-NB is about 50%, and long-term survival remains poor (1, 7). In fact, NB makes up for only 8% of childhood malignant diseases, yet it accounts for 15% of pediatric cancer-related deaths (8). As metastatic relapse is the most common cause of death (9, 10), innovative and effective systemic treatments are required to reduce the mortality of HR-NB patients.

Through systemic administration, molecular radiotherapy (MRT) targets multiple tumor lesions, including micro-metastases not detectable by imaging (11). The only established MRT for NB is iodine-131 meta-iodobenzylguanidine (^131^I-mIBG), a radiolabeled norepinephrine analogue targeting the norepinephrine transporter (NET) (12). ^131^I-mIBG has been used since the 1980s, but it was only recently evaluated in combination with temozolomide in a phase II trial as second-line therapy in relapsed and refractory HR-NB, resulting in an overall objective response (OR) of 13% and a 2-year event-free survival (EFS) of 17% (13). ^131^I-mIBG was also investigated as first-line therapy in the recently concluded Children’s Oncology Group ANBL 1531 Trial (NCT03126916), the results of which are yet unpublished. Previously reported success rates of ^131^I-mIBG treatment have been variable with usually temporary responses, and treatment intensity is often limited by bone marrow toxicity (14–16).

An emerging MRT alternative for NB is the radiolabeled somatostatin analogue lutetium-177 octreotate (^177^Lu-DOTATATE). ^177^Lu-DOTATATE is approved for the treatment of adult neuroendocrine tumors overexpressing somatostatin receptors (SSTR) (17). The drug binds to somatostatin receptors SSTR1–5 and has highest affinity to SSTR2 (18), which is expressed in over 90% of NB, including metastatic lesions (10). The LuDO-N trial (EudraCT: 2020–00445-36, NCT04903899) (10) is an ongoing, multi-center, phase II trial investigating the efficacy of single-agent ^177^Lu-DOTATATE in relapsed or refractory HR-NB. Building on the experience from the previous phases I–IIa LuDO trial (14), which showed stable disease (SD) in five out of six patients but no objective responses, the LuDO-N trial implements individualized dosimetry and intensified administration schedule to deliver a maximal radiation dose within 2 weeks to counter the rapid proliferation that often occurs in relapsed NB. By characterizing the absorbed radiation dose in separate tumor lesions and corresponding SSTR2 expression patterns, the trial strives to understand the factors that contribute to ^177^Lu-DOTATATE treatment response and resistance.

## 2 Case description

**Figure 1:**
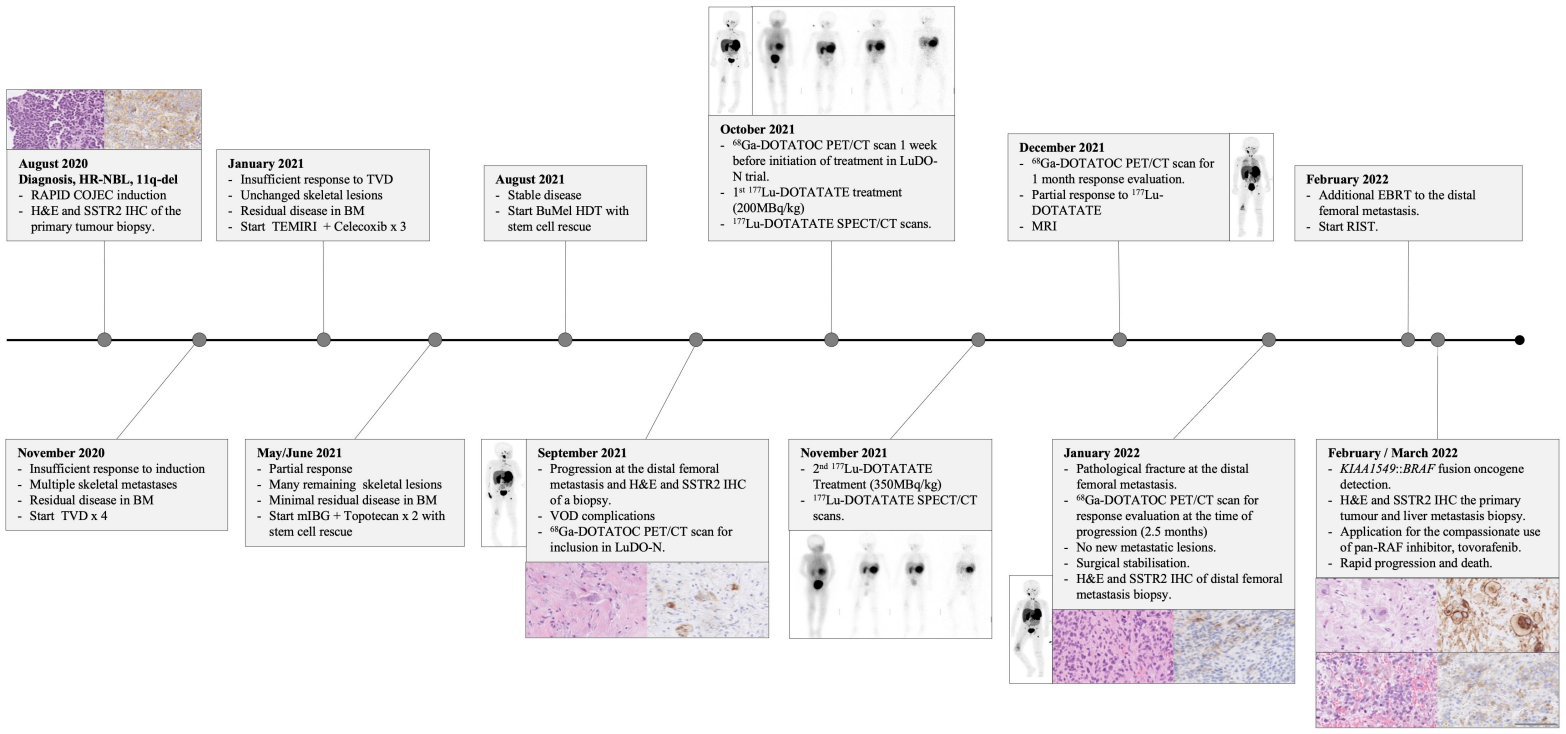
Clinical course timeline.

A 21-month-old, previously healthy girl was diagnosed with HR-NB with widespread metastases. The tumor biopsies displayed poorly differentiated, unfavorable NB, and the urinary catecholamine metabolite levels were elevated. ^123^I-mIBG scintigraphy displayed uptake in the retroperitoneal primary tumor as well as in various metastatic sites, and bone marrow involvement was confirmed by trephines and aspirates. An SNP microarray analysis showed an 11q deletion and no *MYCN* amplification. Induction therapy was initiated according to the HR-NBL1/SIOPEN protocol (3) with Rapid COJEC, followed by four cycles of TVD and further three cycles of TEMIRI with concomitant cyclooxygenase-2 inhibitor celecoxib due to persistent bone marrow involvement. This was followed by ^131^I-mIBG therapy with concomitant topotecan and stem cell rescue prior to high-dose myeloablative chemotherapy with BuMel. Due to progression at a metastatic site in the distal femur, the patient was screened for inclusion in the LuDO-N trial (**Figure 1**).

## 3 Results

### 3.1 Diagnostic assessment of ^177^Lu-DOTATATE therapy

**Figure 2:**
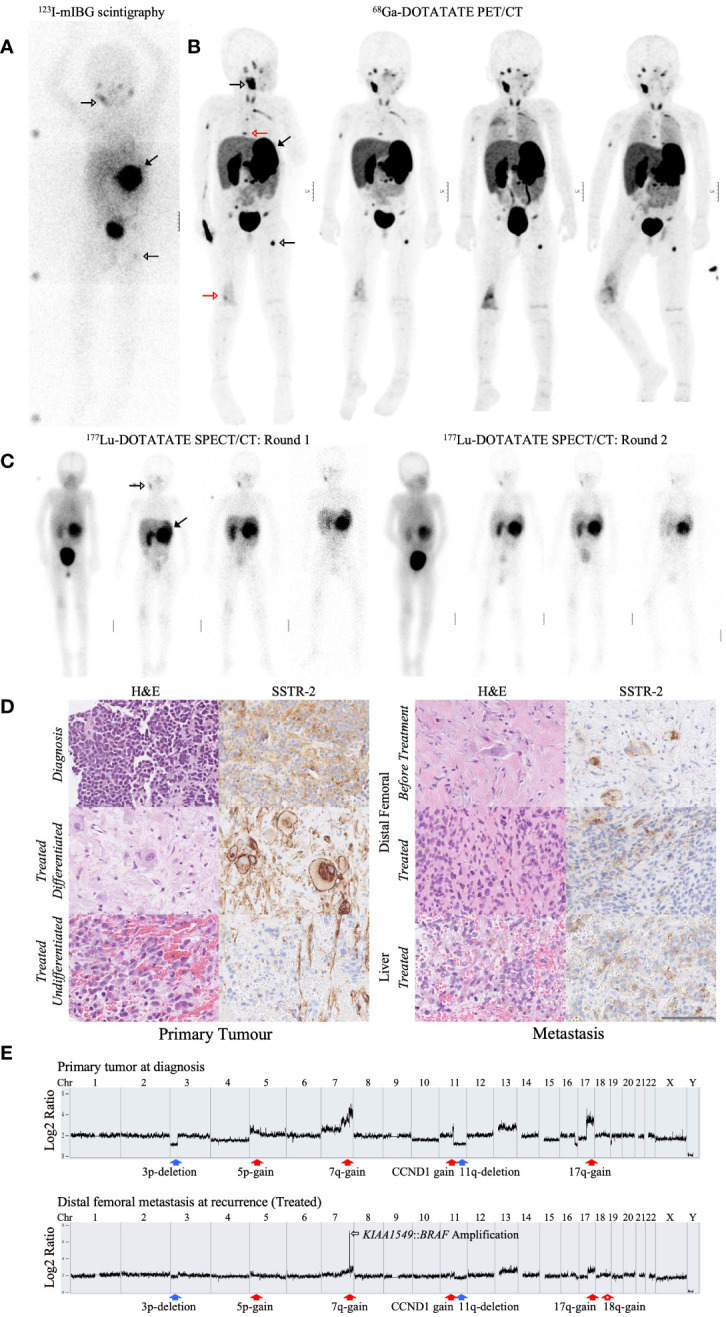
Profiling the radiological, immunohistochemical, and genomic characteristics of the primary tumor and metastatic tumor lesions of a refractory HR-NB patient. **(A)** Diagnostic ^123^I-mIBG SCINT scan taken 3 months after ^131^I-mIBG treatment. **(B)** ^68^Ga-DOTATOC PET/CT scans taken 1 month before, 1 week before, 1 month after, and 2.5 months after the initiation of ^177^Lu-DOTATATE treatment, ordered accordingly from left to right. The ^123^I-mIBG SCINT scan **(A)** and the first ^68^Ga-DOTATOC PET/CT scan **(B)** were taken 1 week apart. Both scans showed uptake in the primary tumor (filled black arrow) and metastatic lesions in the mandible and left femur (empty black arrow). Meanwhile, the ^68^Ga-DOTATOC PET/CT scans were able to detect additional metastatic lesions, including the distal femoral metastasis and a Th7 skeletal metastasis (red arrow). **(C)** Serial ^177^Lu-DOTATATE SPECT/CT scans taken after drug administration. SPECT/CT scans were taken after the first and second fraction of drug administration, respectively. The distribution of ^177^Lu-DOTATATE was visualized over the duration of 3 h, 1 day, 2 days, and 6 days, ordered accordingly from left to right. ^177^Lu-DOTATATE uptake was seen in predominantly the primary tumor (filled arrow) and the mandibular metastasis (empty arrow). **(D)** Hematoxylin and eosin (H&E) staining and immunohistochemical analysis of SSTR2 in the primary tumor and metastatic tumor lesions before and after ^177^Lu-DOTATATE treatment. The primary tumor biopsies were collected at diagnosis and 4 months after ^177^Lu-DOTATATE treatment (Treated). The treated primary tumor displayed both regions of differentiated as well as undifferentiated neuroblastoma cells, as shown by the presence and lack of differentiated ganglioneuroma, respectively. Distal femoral metastasis collected 1 month before starting ^177^Lu-DOTATATE therapy (Before Treatment) and 2.5 months after the end of ^177^Lu-DOTATATE treatment (Treated) from a pathological fracture that developed as the metastasis progressed. Liver metastasis collected together with the treated primary tumor biopsy 4 months after the end of ^177^Lu-DOTATATE treatment (Treated). All sections were imaged at ×40 magnification. The scale bar represents 100 um. **(E)** Cytoscan HD SNP array of the primary tumor at diagnosis and the distal femoral metastasis at recurrence. Both tumor biopsies presented typical chromosomal aberrations associated with HR-NB, including 3p and 11q deletion (filled blue arrows) and 5p, 7q, and 17q gain (filled red arrows). The distal femoral metastasis exhibited a unique *KIAA1549*::*BRAF* fusion amplification at chromosome 7 (empty black arrow) along with 18q gain (empty red arrow). The x-axis represents the genomic position along the chromosome. The y-axis represents the log2 ratio difference in fluorescence intensity from a reference sample DNA.

The patient was included in the LuDO-N trial and received 200 MBq/kg of ^177^Lu-DOTATATE in the first cycle and a further 350 MBq/kg in the second cycle to reach a maximal renal absorbed radiation dose of 23 Gy (**Figure 1**). The ^68^Ga-DOTATOC PET/CT scans, taken before the initiation of ^177^Lu-DOTATATE treatment, showed uptake in the primary tumor and multiple metastatic lesions, many of which were not previously detected by ^123^I-mIBG scintigraphy (**Figures 2A, B**). ^177^Lu-DOTATATE was well tolerated, and toxicity was limited to thrombocytopenia and neutropenia, without a need for stem cell re-infusion. The response evaluation at 1 month showed SD with clinical improvement, and there was a decrease in ^68^Ga-DOTATOC PET/CT scan intensity seen in one skeletal lesion in Th7 (**Figure 2B**) (19); however, known lesions in the abdomen and skeleton remained largely unchanged. The metastatic site in the right distal femur, which had initially progressed, was also stabilized after ^177^Lu-DOTATATE treatment, but 2.5 months after the treatment, the patient developed a pathological fracture. A subsequent response evaluation with ^68^Ga-DOTATOC PET/CT (**Figure 2B**) and MRI (not shown here) confirmed progressive disease (PD) exclusively at this metastatic site. A surgical resection/stabilization was performed, EBRT was given locally, and chemotherapy was initiated according to the RIST protocol. A request for the compassionate use of pan-RAF inhibitor tovorafenib was filed after the detection of a novel *KIAA1549*::*BRAF* fusion amplification in the metastasis; however, the disease progression was rapid, and the patient died before the treatment could be initiated.

### 3.2 Dosimetry in separate tumor lesions

The amount of radiation absorbed by each tumor lesion was calculated from four subsequent ^177^Lu-DOTATATE SPECT/CT scans after each treatment cycle (**Figure 2C**). The uptake of ^177^Lu-DOTATATE resulted in an absorbed dose of 54 Gy in the primary tumor. The dose-limiting risk organ, the kidneys, received a total of 21.4 and 18.3 Gy, to the right and left kidney, respectively, and the total whole-body absorbed dose was 1.58 Gy, adding to the prior ^131^I-mIBG dose of 4 Gy. The metastatic sites in the mandible and left femur received 39 and 21.3 Gy, respectively. In comparison, the metastasis in the distal right femur, which eventually progressed, only received 2 Gy (**Table 1**).

**Table 1:** Dosimetry of tumor lesions and organs-at-risk after two cycles of ^177^Lu-DOTATATE.

	Whole body	Risk organs				Primary tumor	Metastasis		
		Right kidney	Left kidney	Spleen	Liver		Right femur	Left femur	Mandible
**Fraction 1**	0.61	8.6	7.5	6.9	1.5	24.4	0.9	9.1	17.2
**Fraction 2**	0.97	12.9	10.8	7.4	1.3	29.6	1.1	12.2	21.8
**Total AD**	1.58	21.5	18.3	14.3	2.8	54	2	21.3	39

Unit: Gy.

Total AD: total absorbed dose of radiation.

### 3.3 SSTR2 immunohistochemistry of tumor lesions

Biopsies from the primary tumor, distal femoral metastasis, and liver metastasis were collected at different occasions from diagnosis to relapse (**Figure 2D**). The biopsies were stained for H&E and SSTR2. The SSTR2 staining of tumor lesions were quantified using a scoring system adapted from Volante et al. (20). The scoring system accounts for the percentage of SSTR2-positive cells and the subcellular distribution of SSTR2 expression, with emphasis on membranous staining which is clinically relevant for ^177^Lu-DOTATATE uptake (20) (**Table 2**). The treatment-naïve primary tumor sampled at diagnosis displayed cytoplasmic SSTR2 staining in more than 50% of NB cells. The primary tumor sample collected 19 months later (4 months after ^177^Lu-DOTATATE treatment) displayed a heterogeneous SSTR2 expression. Most of the cells had a differentiated, ganglioneuroma-like morphology and strong SSTR2 staining with complete membrane outlining; however, within the same tissue section, there was also a cluster of undifferentiated NB cells, with less than 10% of the cells expressing SSTR2.

**Table 2:** SSTR2 IHC scoring of the primary tumor and metastatic tumor lesions. IHC SSTR2 staining for the same tumor lesions as in **Figure 2D** were quantified for SSTR2 expression. The “Timeline” column indicates the number of months that have passed since diagnosis and the end of ^177^Lu-DOTATATE treatment (“Treatment”). The “Morphology” column describes the state of differentiation of the NB cells. The “Scoring” column scores the SSTR2 expression from 0 to 3 based on the localization of the SSTR2 staining and the percentage of cells that are positive for SSTR2 in the tissue. The “Overall” column is the sum of the “Localization” and the “Percentage positive cells” column.

	Timeline (months)		Morphology	Scoring		
	Diagnosis	Treatment		Localization	Percentage positive cells	Overall
**Primary tumor** (diagnosis)	0	-14	Undifferentiated, hyperchromatic cells	1	3	4
**Primary tumor** (treated, differentiated)	19	4	Predominantly differentiated, ganglioneuroma-like cells	3	3	6
**Primary tumor** (treated, undifferentiated)	19	4	Undifferentiated cells	2	0	2
**Right femoral metastasis** (before treatment)	13	-1	Undifferentiated cells, varying degrees of differentiation	1	0	1
**Right femoral metastasis** (treated)	17	2.5	Undifferentiated cells	1	1	2
**Liver metastasis** (treated)	19	4	Undifferentiated cells	2	1	3

Treated: after treatment with ^177^Lu-DOTATATE, Before Treatment: before treatment with ^177^Lu-DOTATATE.

Localization: 0: no staining, 1: diffuse or focal cytoplasmic staining, 2: incomplete membrane-associated staining with or without cytoplasmic staining, 3: complete membrane-associated staining.

Percentage of positive cells: 0, 0%–10% positive cells; 1, 11%–25% positive cells; 2, 26%–50% positive cells; 3, >50% positive cells.

The metastatic tumor lesions also displayed a heterogeneous SSTR2 expression. The liver metastasis, which was collected 4 months after ^177^Lu-DOTATATE treatment, showed cytosolic staining with partial membranous staining in about 25% of NB cells. The biopsies from the right distal femoral metastasis, collected 1 month before ^177^Lu-DOTATATE and at relapse 2.5 months after ^177^Lu-DOTATATE, showed a consistently weak SSTR2 staining with undifferentiated NB cell morphology. Both samples exhibited a predominantly cytosolic staining with a partial membranous staining in the post-treatment biopsy (**Table 2**).

### 3.4 Genomic profile of tumor lesions

Cytoscan HD SNP microarray was performed on the treatment-naïve primary tumor at diagnosis as well as on the distal femoral metastasis at the time of recurrence, which was 17 months after diagnosis (**Figure 2E**). The genomic profiles of both tumor lesions were consistent with HR-NB and showed several persistent segmental alterations, including 3p and 11q deletion, gain of *CCND1*, 7q, 17q, and an alteration at 5p indicating *TERT* juxtaposition. In the distal femoral metastasis, a unique focal amplification of fusion oncogene *KIAA1549*::*BRAF* was present at chromosome 7 as well as 18q gain (**Supplementary Table S1**). Additionally, interphase fluorescence *in situ* hybridization (FISH) analysis confirmed *BRAF* amplification in the distal femoral metastasis which was not seen in the liver metastasis collected at the same timepoint (**Supplementary Figure S1A**).

## 4 Discussion

We report the first patient case in the LuDO-N trial, investigating the therapeutic potential of single-agent ^177^Lu-DOTATATE in refractory or relapsed HR-NB (10). The patient presented with metastatic HR-NB, insufficient response during first-line induction therapy, and disease progression during second-line therapy, and she was thereon included in the LuDO-N trial. After ^177^Lu-DOTATATE treatment, the patient presented a mixed treatment response with initial SD, followed by disease progression at a single metastatic site (**Figure 1**). We analyzed the SSTR2 expression patterns in tumor biopsies collected throughout the course of the disease and correlated SSTR2 expression with absorbed radiation doses and treatment response in the separate tumor lesions.

An immunohistochemical analysis demonstrated heterogeneity of SSTR2 expression in the tumor lesions. The treatment-naïve primary tumor tissue exhibited a relatively high expression of SSTR2 (**Figure 2D**; **Table 2**). After treatment, the primary tumor displayed clonal heterogeneity, with a cluster of undifferentiated NB cells with low SSTR2 expression among a majority of differentiated, ganglioneuroma-like cells with a high SSTR2 expression. The ganglioneuroma-like cells suggest radiation-induced morphological maturation of NB cells, an observation previously demonstrated in mouse (21) and human NB cells (22). Additionally, others have also reported radiation-induced cellular maturation through the activation of signaling pathways in cancer (23) and neural stem cells (24, 25). Clonal heterogeneity and low SSTR2 expression in the treated primary tumor may explain the lack of disease regression on imaging despite absorbing a substantial radiation dose of 54 Gy (**Table 1**).

Metastatic tumor lesions consistently exhibited weak SSTR2 staining and predominantly undifferentiated NB cell morphology (**Figure 2D**; **Table 2**). The distal femoral metastasis, which eventually progressed, initially exhibited a purely cytoplasmic SSTR2 expression before treatment and shifted to a partial membranous staining after treatment. This shift may result from radiation-induced upregulation of SSTR2 expression observed in different cancer models, resulting in a higher uptake of radiolabeled somatostatin analogues (26–29). This slight increase in absorbed radiation in the second fraction of ^177^Lu-DOTATATE administration supports this (**Table 1**). Despite this improvement, the distal femoral metastasis was only able to absorb a total of 2 Gy, suggesting that the local progression in this metastatic site may be a consequence of poor ^177^Lu-DOTATATE uptake due to low SSTR2 expression.

The novel, focal amplification of the oncogenic fusion *KIAA1549*::*BRAF* in the progressed distal femoral metastasis suggests a potential source of resistance to ^177^Lu-DOTATATE therapy. This fusion is expected to cause constitutive activation of the RAS/MAPK signaling pathway, promoting tumorigenesis and treatment resistance through mechanisms such as enhanced cell survival and upregulation of drug efflux transporters (30–32). Dysregulation of the RAS-MAPK pathway is frequent in relapsed NB (33–35), although *BRAF* mutations are rare and detected in <1% (36). Furthermore, neither *KIAA1549*::*BRAF* nor the breakpoints at exons 10;10 (**Supplementary Figure S1D**) have been described in NB, making this report the first observation of its kind.

The *KIAA1549*::*BRAF* fusion has been described in pilocytic astrocytoma, where it is associated with a favorable prognosis (37), and in pediatric low-grade astrocytoma (38). However, clinical implications in NB and potential contribution to ^177^Lu-DOTATATE treatment resistance remain to be understood. Nonetheless, this fusion presents a therapeutic vulnerability that can be strategically addressed through targeted therapy such as the pan-RAF inhibitor tovorafenib, for which we did apply for compassionate use. This approach mirrors the successful use of ALK inhibitors in *ALK*-mutated NB (39, 40), highlighting the potential of molecular profiling to identify actionable genetic alterations for targeted therapies.

The heterogeneous expression of SSTR2 in NB patients has been reported before. Immunohistochemical analyses conducted on large cohorts of tumor samples observed higher SSTR2 expression in differentiated and low- and intermediate-risk NB than in HR-NB (41, 42). Furthermore, Gains et al. (42), also observed clonal heterogeneity in their patients’ samples and proposed the use of a combination of radionuclides for better radiation coverage of heterogeneous tumor regions. The heterogeneity in SSTR2 expression seen in this case report, as well as by others, highlights the importance of understanding the potential impact of SSTR2 expression on ^177^Lu-DOTATATE treatment outcomes. Continued analyses in the LuDO-N trial will further elucidate this correlation and aid in predicting treatment response and guiding treatment strategies. Additionally, exploring alternative treatment approaches, such as SSTR2 upregulation (28, 43) and the use of radiosensitizers (44, 45), may be beneficial to improve the outcomes for HR-NB patients with a mixed response to ^177^Lu-DOTATATE.

This study provides early insights from the LuDO-N trial and highlights the correlation of clonal heterogeneity and a mixed response to ^177^Lu-DOTATATE therapy in a patient with refractory HR-NB. Disease progression in the distal femoral metastasis revealed heterogeneous SSTR2 expression patterns which were reflected by low uptake of radiation in dosimetry analysis. Furthermore, genotyping uncovered a novel *KIAA1549*::*BRAF* fusion gene amplification, potentially accelerating disease progression at the metastatic site. These findings warrant further investigations into the correlation between SSTR2 expression and ^177^Lu-DOTATATE treatment outcomes and the clinical implications of *KIAA1549*::*BRAF* amplification in NB. As the success of MRT ultimately depends on the adequate delivery of radiation to all malignant cells, clonal heterogeneity remains a challenge. A successful treatment for advanced NB may require multi-agent therapy tailored to distinct tumor clones and escape mechanisms within each patient. Bringing complimentary targeted therapies forward to frontline therapy could, at least to some extent, reduce the detrimental effect of ongoing clonal evolution and improve the possibilities to ultimately achieve cure.

## 5 Patient’s perspective

During treatment with ^177^Lu-DOTATATE, the patient experienced flushing that resolved spontaneously within 10 min, but the treatment was generally well tolerated. The radiation levels decreased rapidly, and the family spent only 24 h in isolation during each of the two courses of treatment. The pre-treatment baseline ^68^Ga-DOTATOC PET as well as the post-treatment serial SPECT/CT dosimetry scans required altogether seven procedures under general anesthesia during a 3-week period. The patient was hospitalized for a total of 3 days during each of the two treatment sessions but was discharged and stayed at home between the treatments.

Two adverse events occurred during the first 30 days post-treatment: when the patient was hospitalized due to hematemesis secondary to epistaxis, which was resolved with thrombocyte and erythrocyte transfusions, but without further interventions. During one of these episodes, she also tested positive for a COVID-19 infection. Apart from this, the patient recovered steadily from the ^177^Lu-DOTATATE treatment. The blood thrombocyte and neutrophil levels decreased initially, but a re-infusion of stem cells was never required. At follow-up 1 month after treatment, she had completely recovered, and the pain in her right lower limb, which she had initially before inclusion, had resolved. Unfortunately, this lasted only until 2.5 months after treatment when she was re-hospitalized for a pathological fracture secondary to disease progression.

## 6 Methods

### 6.1 Dosimetry

The absorbed doses for tumor lesions and organs-at-risk were calculated based on four SPECT/CT acquisitions after each ^177^Lu-DOTATATE administration. The absorbed doses to organs-at-risk (liver, kidneys, and spleen) were calculated using a small region of interest to estimate the average activity concentration in the organ, as described by Sandström et al. (46). This method was also applied to tumor lesions >2 cm in diameter with a homogeneous activity distribution. For smaller tumor lesions, a water dose-kernel method was used. For dose-kernel-based calculations, images were convoluted with the dose point-kernel. The regions of interest and recovery coefficients were based on tumor sizes determined on the baseline PET/CT scan. Whole-body absorbed doses were calculated using whole-body scintigraphy. The time–activity curves were integrated using trapezoidal (first 25 h after administration) and exponential (25 h and later) curve fits. The cumulated activities were multiplied with age-adjusted S-values extracted from Olinda software.

### 6.2 Immunohistochemistry

Immunohistochemistry on histological material was performed as part of clinical routine. Positively charged slides with 4-μm FFPE tissue sections were stained with anti-somatostatin receptor 2 antibody (UMB1 clone, #ab134152, RRID: AB_2737601, Abcam) using the fully automated Ventana BenchMark Ultra. A *post-hoc* analysis of SSTR2 IHC was performed on the primary tumor, liver metastasis, and the distal femoral metastasis that eventually progressed.

The expression level of SSTR2 was quantified using a scoring system adapted from Volante et al. (20). SSTR2 localization and the percentage of positive cells were quantified on a scale of 0–3. The resulting values were summed up to create an overall score ranging from 0 to 6. Each slide was scored by two independent reviewers. The scoring criteria and their interpretations were as follows:

SSTR2 localization score:

- 0: No staining.
- 1: Diffuse or focal cytoplasmic staining.
- 2: Incomplete membrane-associated staining with or without cytoplasmic staining.
- 3: Complete membrane-associated staining.

Percentage of SSTR2-positive cell score:

- 0: 0%–10%.
- 1: 11%–25%.
- 2: 26%–50%.
- 3: >50%.

Overall score: The localization score and the percentage of positive cell score were combined for the overall score.

Interpretation of the overall score:

- Score 0: No expression.
- Scores 1–2: Low expression (diffuse or focal cytoplasmic staining and/or low percentage of positive cells).
- Scores 3–4: Moderate expression (cytoplasmic staining or incomplete membrane-associated staining and/or moderate percentage of positive cells).
- Scores 5–6: High expression (complete membrane-associated staining and/or high percentage of positive cells).

### 6.3 Genomics profile with SNP microarray

Microarray analyses of DNA from tumor were performed using Affymetrix Human Cytoscan High Density arrays (Affymetrix, Thermo Fisher, Waltham, MA, USA) essentially as described previously (47). The GDAS software (Affymetrix) was used for primary data analysis, while genomic profiles were generated using Chromosome Analysis Suite (ChAS 3.3; Thermo Fisher Scientific).

### 6.4 Whole-genome sequencing

The procedures for WGS with consecutive bioinformatical analyses and filtering procedures have been described in detail earlier (33). Briefly, paired-end sequencing was performed on Illumina instrumentation at Clinical Genomics, The Science for Life Laboratories, Gothenburg, Sweden, for an average coverage of 161X, 152X, and 35X for DNA from tumor material at the time of diagnosis, metastasis in the femur at the time of recurrence, and from blood lymphocytes, respectively. Mapping and variant calling were performed using the Sentieon suite of bioinformatical tools (Sentieon Inc, Mountain View, CA, USA) together with the identification of copy number variants through the Canvas tool (48), while structural variants were called using Manta (49), keeping only the variants supported by both paired and split reads. Additional SNV filtering was done by keeping only the so-called high-quality nonsynonymous variants, including variants in canonical splice sites, with coverage above 10 and gnomAD allele frequency below 3%.

### 6.5 Fluorescence *in situ* hybridization

Interphase FISH analysis was performed on imprint slides from the liver metastasis as well as on formalin-fixed, paraffin-embedded tissue sections from the distal femoral metastasis. The pretreatment procedures for the formalin-fixed material were as recommended by Abbott, Vysis (Vysis Inc., Downers Grove, IL, USA). Sections of 2 to 5 μm were hybridized with a dual-color break apart probe for the gene *BRAF* (7q34) ZytoLight (Zytovision, GmbH, Bremerhafen, Germany). The imprint slides were also treated as recommended by Abbott, Vysis. All slides were counterstained with 4′,6′-diamidino-2′-phenylindole dihydrochloride (DAPI) and evaluated using a Zeiss Imager 22 Imaging fluorescence microscope. The slides were analyzed and re-analyzed by two independent reviewers. The interpretation of intact, fusion, split signals, and amplification was based on accepted international guidelines.

## Data Availability

The datasets for this article are not publicly available due to concerns regarding participant/patient anonymity. Requests to access the datasets should be directed to the corresponding author.
