# Supplementary material for: Heterogeneous SSTR2 target expression and a novel KIAA1549::BRAF fusion clone in a progressive metastatic lesion following 177Lutetium-DOTATATE molecular radiotherapy in neuroblastoma: a case report
Source: Front Oncol. 2024 Sep 11;14:1408729. doi: 10.3389/fonc.2024.1408729 (PMC11422106; doi:10.3389/fonc.2024.1408729)
Supplement: Supplementary file 1 [file DataSheet1.docx]

Supplementary Material

Heterogeneous SSTR2 target expression and a novel *KIAA1549*::*BRAF* fusion clone in a progressive metastatic lesion following ^177^Lutetium-DOTATATE molecular radiotherapy in neuroblastoma: A Case Report

**Se Whee Sammy Park^1*^, Susanne Fransson^2^, Fredrik Sundquist^3,4^, Joachim N. Nilsson^5,6^, Per Grybäck^5,6^, Sandra Wessman^1,7^, Jacob Strömgren^8^, Anna Djos^2^, Henrik Fagman^2^, Helene Sjögren^9^, Kleopatra Georgantzi^4,10^, Nikolas Herold^4,10^, Per Kogner^4,10^, Dan Granberg^11^, Mark N. Gaze^12^, Tommy Martinsson^2^, Kasper Karlsson^1†^ and Jakob J. E. Stenman^4,13†^**

^1^ Department of Oncology-Pathology, Karolinska Institutet, Stockholm, Sweden

^2^ Department of Laboratory Medicine, Sahlgrenska Academy, University of Gothenburg, Gothenburg, Sweden

^3^ Department of Urology, Karolinska University Hospital, Stockholm, Sweden

^4^ Department of Women’s and Children’s Health, Karolinska Institutet, Stockholm, Sweden

^5^ Department of Medical Radiation Physics and Nuclear Medicine, Karolinska University Hospital, Stockholm, Sweden

^6^ Department of Molecular Medicine and Surgery, Karolinska Institutet, Stockholm, Sweden

^7^ Department of Pathology and Cancer Diagnostics, Karolinska University Hospital, Stockholm, Sweden

^8^ Department of Pediatric Radiology, Karolinska University Hospital, Stockholm, Sweden

^9^ Clinical Genetics and Genomics, Sahlgrenska University Hospital, Gothenburg, Sweden

^10^ Department of Pediatric Oncology, Karolinska University Hospital, Stockholm, Sweden

^11^ Department of Breast, Endocrine Tumors and Sarcomas, Department of Molecular Medicine and Surgery, Karolinska University Hospital, Karolinska Institutet, Stockholm, Sweden

^12^ Department of Oncology, University College London Hospitals NHS Foundation Trust, London, United Kingdom

^13^ Department of Pediatric Surgery, Karolinska University Hospital, Stockholm, Sweden

**^†^ Equal contribution**

*** Corresponding Author**

# Supplementary Figures and Tables

## Supplementary Figures

##
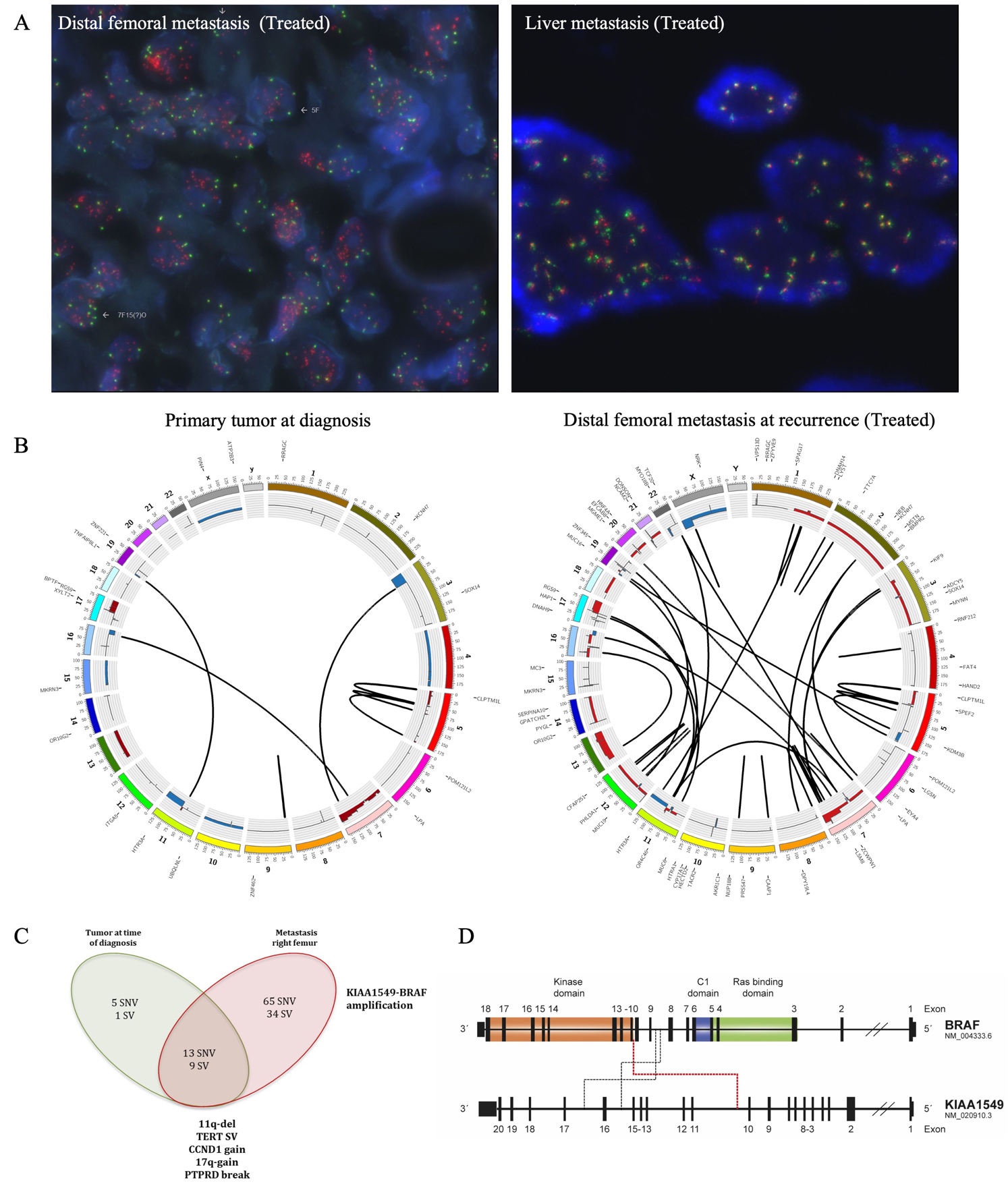


**Supplementary Figure 1. Genomic profiling of the of the primary tumor at diagnosis and the distal femoral metastasis at recurrence. (A)** FISH analysis using a dual break apart probe showed high and specific amplification of the 3´-part of BRAF (red) together with 2-17 copies 5´-part of BRAF (green) in the distal femoral metastasis, but minimal colocalization, supporting the *KIAA1549*::*BRAF* fusion amplification (left panel). No specific amplification of the 3'-part of BRAF was seen in the liver metastasis sampled at the same occasion since the probes colocalized. However, 7-15 copies of intact BRAF were identified, due to 7q copy number gain (right panel). (**A**) Circos plots showing structural variants (SV), copy number alterations and somatic single nucleotide variants (SNVs) for the primary tumor at diagnosis and the distal femoral metastasis at recurrence (Treated). Copy number plots calculated on coverage ratio between tumor and corresponding normal tissue (blood lymphocytes) are shown on the inner circle with gain of genomic material indicated in read and loss of genomic material indicated in blue. The lines within the inner circle indicate SVs within and between chromosomes while genes affected by somatic SNVs are shown on the outer circle. (**C**) Distribution of SNVs and SVs either unique or shared between the two tumor biopsies. (**D**) The fusion with break in *KIAA1549* intron 10 and in *BRAF* in intron 10, indicated by red line, is expected to be in frame as judged by sequence data. The two most common fusion points for *KIAA1549*::*BRAF* fusions in pilocytic astrocytoma are indicated by the black lines.

## Supplementary Table

**Supplementary Table 1. Whole-genome sequencing statistics, somatic non-synonymous single nucleotide variants and structural variants for the primary tumor and the distal femoral metastasis at recurrence.**

*File attached as .xlsx format.*

## Supplementary Table 2. Abbreviations.

Rapid COJEC: Cisplatin, vincristine, carboplatin, etoposide, and cyclophosphamide

H&E: Hematoxylin and eosin histochemical staining

SSTR2: Somatostatin receptor 2

TVD: Topotecan, vincristine and doxorubicin

BM: Bone marrow

TEMIRI: Temozolomide and irinotecan

mIBG: ^123^Iondine meta-Iodobenzylguanidine

BuMel: Busulfan and melphalan hydrochloride

IHC: Immunohistochemistry

HDT: High dose chemotherapy

VOD: Veno-occlusive disease

EBRT: External beam radiotherapy

RIST: Irinotecan, temozolide, sirolimus and dasatinib.

PET/CT: Positron emission tomography / computed tomography

SPECT/CT: Single photon emission computed tomography / computed tomography

FISH: Fluorescent *in situ* hybridization

SD: Stable disease

PD: Progressive disease
